# TUG1 promotes prostate cancer progression by acting as a ceRNA of miR-26a

**DOI:** 10.1042/BSR20180677

**Published:** 2018-10-02

**Authors:** Bin Yang, Xiaodi Tang, Zhixin Wang, Daju Sun, Xin Wei, Youpeng Ding

**Affiliations:** 1 Department of Breast Surgery, China-Japan Union Hospital of Jilin University, Changchun 130033, P.R. China; 2 Department of Radiation Oncology, China-Japan Union Hospital of Jilin University, Changchun 130033, P.R. China; 3 Department of Urology, China-Japan Union Hospital of Jilin University, Changchun 130033, P.R. China; 4 Department of Pathology, China-Japan Union Hospital of Jilin University, Changchun 130033, P.R. China

**Keywords:** EMT, LncRNA, metastasis, miR-26a, PCa, TUG1

## Abstract

Previous studies have demonstrated that taurine-upregulated gene 1 (TUG1) was aberrantly expressed and involved in multiple types of cancer; however, the expression profile and potential role of TUG1 in prostate cancer (PCa) remains unclear. The aim of the present study was to evaluate the expression and function of TUG1 in PCa. In the present study, we analyzed TUG1 expression levels of PCa patients in tumor and adjacent normal tissue by real-time quantitative PCR. Knockdown of TUG1 by RNAi was performed to explore its roles in cell proliferation, migration, and invasion. Here we report, for the first time, that TUG1 promotes tumor cell migration, invasion, and proliferation in PCa by working in key aspects of biological behaviors. TUG1 could negatively regulate the expression of miR-26a in PCa cells. The bioinformatics prediction revealed putative miR-26a-binding sites within TUG1 transcripts. In conclusion, our study suggests that long non-coding RNA (lncRNA) TUG1 acts as a functional oncogene in PCa development.

## Introduction

Prostate cancer (PCa) is the second most frequently diagnosed cancer in men that leads to second or third cancer-related deaths worldwide [1]. Despite improved therapy concept and method, the recurrence of PCa within 5 years remains approximately 25% [2]. Metastasis and invasion are the main causes of these lethal consequences of PCa [3]. Thus, deciphering the mechanism of invasive and metastatic behavior are of great importance for early diagnosis and therapy of PCa.

Long non-coding RNAs (lncRNAs) are important new members of the family of ncRNAs with limited or no protein-coding capacity [4,5]. Cumulative evidence is emerging that lncRNAs impact the biological functions of many different cancer types, including PCa [6–8]. Recent studies have shown that the biological behavior of the tumor could be regulated by specific lncRNA factors [9]. Taurine-upregulated gene 1 (TUG1) is a 7.1-kb lncRNA, which was initially identified as a transcript that up-regulated in response to taurine treatment of developing mouse retinal cells [10]. Recently, TUG1 has been found to be elevated in a number of cancers, including head and neck, gallbladder, lung, and pancreatic cancer [11–13]. However, limited data are available for the molecular mechanisms of TUG1 in PCa, largely due to a lack of specific investigation. In order to evaluate whether PCa cells would be sensitive to TUG1 blockade, we showed that TUG1 is overexpressed in PCa, that TUG1 inhibition increased cell apoptosis, that TUG1 knockdown in PCa cells resulted in a significant decrease in cell growth, migration, and invasion.

## Materials and methods

### Patients and specimens

The present study included 86 consecutive men diagnosed with PCa affirmed by clinical resection and pathology during 2009–2014. Cancer tissues and adjacent normal tissues surgically removed from PCa patients were immediately frozen in liquid nitrogen and stored at −80°C. The present research was sanctioned by the Institutional Research Ethics Committee of China-Japan Union Hospital of Jilin University, and informed consent was signed by all the 86 patients.

### Cell lines

RWPE1 and PCa cell lines, DU145, PC-3, LNCaP, and 22Rv1, were purchased from American Type Culture Collection (ATCC, Rockville, MD). PCa cells were cultured in RPMI-1640 or minimum essential Eagle’s medium, supplemented with 10% FBS and antibiotics. The human non-tumorigenic prostate epithelial cell line RWPE-1 was cultured in keratinocyte serum-free medium supplemented with 5 ng/ml human recombinant epidermal growth factor and 30 mg/ml bovine pituitary extract (Invitrogen, Carlsbad, CA). Cultures were maintained in a 5% CO_2_ humidified atmosphere at 37°C.

### RNA preparation and quantitative real-time PCR

Total RNA from tissues or whole-cell lysates was isolated using TRIzol (Life Technologies, Carlsbad, CA, U.S.A.). For quantitative real-time PCR (qRT-PCR), cDNA was synthesized with the PrimeScript RT Master Mix (Takara, Dalian, China) from 500 ng of RNA. The real-time PCR analyses were performed using SYBR Premix Ex Taq II (Takara).

### siRNA

Two siRNAs against TUG1 (si-TUG1) at different sites and one negative control (si-NC) with no definite target were employed and synthesized by GenePharma (Shanghai, China). Cells were seeded on six-well plates at a density of 3 ×  10^5^/well overnight, and then transfected with siRNA or the negative control at a final concentration of 100 nM using Lipofectamine 2000 (Invitrogen, U.S.A.). Forty-eight hours after transfection, the cells were harvested to detect the overexpression or knockout efficiency via qRT-PCR. The sequences of the three designed IncRNA TUG1 siRNAs were as follows: si-TUG1 1#, CAGUCCUGGUGAUUUAGACAGUCUU; si-TUG1 2#, and CCCAGA AGUUGUAAGUUCACCUUGA.

### CCK-8 assay

The proliferation of PCa cells was tested by CCK-8 kit (Dojindo, Japan). Approximately transfected 3.5 × 10^3^ cells in 100 ml were incubated in triplicate in 96-well plates. Following 48 h incubation at 37°C in a humidified atmosphere containing 5% CO_2_, the CCK-8 reagent (10 ml) was added to each well and incubated for another 2 h. The optical density at 450 nm was measured using FLx 800 Fluorescence Microplate Reader (Biotek).

### Flow cytometric analysis

Cells were harvested directly or 48 h after siRNA transient transfection and washed with ice-cold PBS. The PI/RNase staining kits (Multisciences, Hangzhou, China) and Annexin V- FITC Apoptosis Detection Kits (KeyGEN Biotech, Nanjing, China) were used to detect cell cycle and apoptosis in an FACScan instrument (Becton Dickinson, Mountain View, CA, U.S.A.), respectively.

### Transwell migration/invasion assay

Transwell chamber was used to measure cell migration and invasion abilities. In brief, culture inserts with 8-mm pore size (Transwell; Corning, NY) were placed into 24-well plates. Before the measurement of invasion ability, the plates were precoated with matrigel. Two hours before the addition of matrigel, 500 µl of serum-free medium was independently added to the upper and lower chambers, followed by incubation at 37°C for hydration. Cells were digested by trypsin, and resuspended in serum-free medium. The cell density was adjusted to 1 × 10^5^/ml. Then, 200 µl of cell suspension was added into the upper chamber, and 500 µl of DMEM containing 10% FBS into the lower chamber. After incubation at 37°C with 5% CO_2_ for 24 h, the Transwell chamber was removed, cells were washed with 1× PBS, fixed in paraformaldehyde for 20 min, and then stained with 0.1% Crystal Violet for 20 min. The cotton swab was used to clean the non-migrated cells in the upper chamber, cells migrating through the membrane were counted in five randomly selected fields under a microscope (Nikon) at a magnification of ×100.

### Western blot analysis

Proteins were quantitated by Bradford method. Then, total protein extracts was fractionated by 10% SDS/PAGE and transferred to PVDF membranes (GE Healthcare, Piscataway, NJ, U.S.A.). The membranes were blocked with 5% milk at room temperature for 2 h. The primary antibodies, including anti-E-cadherin, anti-N-cadherin, anti-Vimentin (Santa Cruz Biotechnology, Santa Cruz, CA, U.S.A.), and anti-GAPDH antibody (Cell Signaling Technology), were added and incubated with blots at 4°C for 12 h. Target protein bands were visualized using the ECL method.

### Luciferase reporter assay

Cells were seeded in 96-well plates at a density of 5 × 10^3^ cells per well 24 h before transfection. The cells were co-transfected with a mixture of miR-26a or miR-control and wild-type or mutant TUG1 fragment, using Lipofectamine 2000 (Invitrogen). After 48 h, the luciferase activity was measured with a dual luciferase reporter assay system (Promega, Madison, WI). The relative luciferase activity was normalized against the *Renilla* luciferase activity.

### Statistical analysis

Continuous variables were expressed as means ± S.D.; differences were assessed for the significance of using Student’s *t* test or the Mann–Whitney test. Categorical variables were evaluated using chi-square or Fisher exact tests, as appropriate. A *P*-value <0.05 was considered statistically significant. All tests were performed on SPSS Statistics 20.0 software.

### Informed consent

Informed consent was obtained from all individual participants included in the study.

Research involving patients: all procedures performed in studies involving humans were in accordance with the ethical standards of the institution or practice at which the studies were conducted.

The datasets used and/or analyzed during the current study are available from the corresponding author on reasonable request.

## Results

### Expression of TUG1 and miR-26a in PCa tissue samples

**Figure 1:**
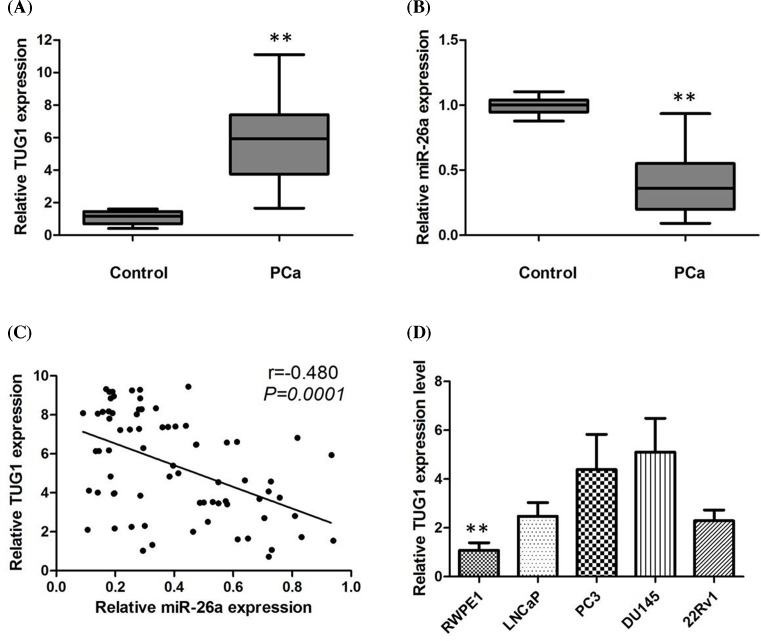
Expression of TUG1 and miR-26a in PCa tissue samples (**A**) qRT-PCR showing expression level of TUG1 in PCa tissues and adjacent non-cancerous tissues; (**B**) qRT-PCR showing expression level of miR-26a in PCa tissues and adjacent non-cancerous tissues; (**C**) miR-26a expression was negatively associated with TUG1 expression in PCa tissues; (**D**) qRT-PCR showing expression level of TUG1 in PCa cell lines. All tests were performed at least three times. Data were expressed as mean ± S.D.; **P<0.01.

We used qRT-PCR assay to study the TUG1 expression in primary PCa tissues. Expression of TUG1 in PCa tissues is significantly higher than that of in normal paired tissues (Figure 1A). Then, we used the qRT-PCR assay to detect the expressions of miR-26a in the PCa tissues. When compared with normal tissues, the miR-26a expression reduced significantly in the PCa tissues (*P*<0.05; Figure 1B), and miR-26a expression was negatively associated with TUG1 expression in PCa tissues (Figure 1C).

### TUG1 promoted PCa cells proliferation *in vitro*

**Figure 2:**
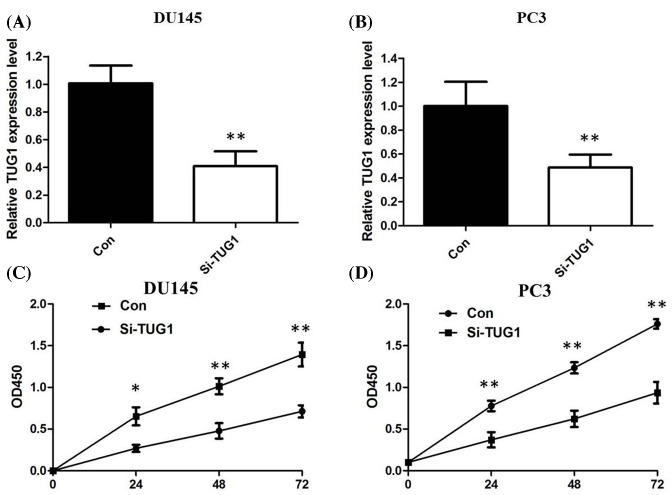
siRNA was employed to knockdown TUG1 and influence of TUG1 on cellular proliferation (**A**,**B**) We employed siRNA to enhance efficiency of TUG1 knockdown in PCa cell lines; (**C**) CCK8 assay showing knockdown of TUG1 inhibited cell proliferation of DU145 cells; (**D**) CCK8 assay showing knockdown of TUG1 inhibited cell proliferation of PC3 cells. All tests were performed at least three times. Data were expressed as mean ± S.D.; *P<0.05, **P<0.01.

TUG1 was highly expressed in diverse PCa cell lines, including LNCaP, DU145, PC3, and 22Rv1, as compared with that in an immortalized non-tumorigenic human prostate epithelial cell line RWPE1 cells, a finding confirmed by qRT-PCR assays (*P*<0.05; Figure 1D). DU145 and PC3 cell lines have the highest levels of TUG1. To evaluate the effectiveness of TUG1 in PCa, silencing of TUG1 by siRNA was performed in DU145 and PC3 cells. The qPCR assays revealed that TUG1 expression was significantly reduced in DU145 and PC3 cell lines (*P*<0.05; Figure 2A,B). There was a significant decrease in proliferation both in DU145 and PC3 cells after knockdown of TUG1 determined by CCK8 (*P*<0.01; Figure 2C,D).

### Knockdown of TUG1 induced PCa cells apoptosis

**Figure 3:**
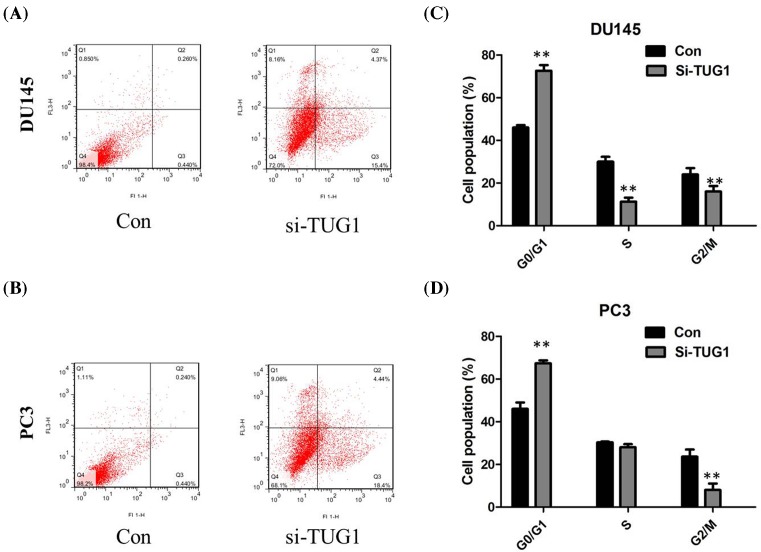
Influence of TUG1 on cell apoptosis and cell cycle (**A**) Flow cytometry showed that DU145 cells transfected with TUG1 siRNA had higher apoptotic rate in comparison with control cells; (**B**) flow cytometry showed that PC3 cells transfected with TUG1 siRNA had higher apoptotic rate in comparison with control cells; (**C**) DU145 cells transfected with si-TUG1 had cell-cycle arrest at the G_1_-G_0_ phase; (**D**) PC3 cells transfected with si-TUG1 had cell-cycle arrest at the G_1_-G_0_ phase; all tests were performed at least three times. Data were expressed as mean ± S.D.; **P<0.01.

To further determine whether the effect of TUG1 on PCa cells proliferation reflected cell apoptosis, we performed flow cytometry. The results showed that DU145 and PC3 cells transfected with TUG1 siRNA had higher apoptotic rate in comparison with control cells (Figure 3A,B). Experiments using siRNA confirmed the effects of the knockdown of TUG1 on cell arrest. Flow cytometric analysis revealed that the knockdown of TUG1 resulted in cell arrest in G_1_ phase of cell cycle; both in DU145 and PC3 cell lines (Figure 3C,D).

### TUG1 promoted PCa cell invasion via regulating EMT

**Figure 4:**
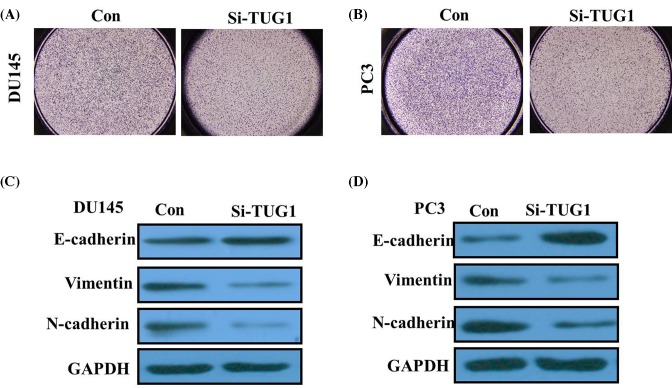
TUG1 regulates cell migration and invasion of PCa (**A**) Inhibition of invasion of DU145 cells by TUG1 siRNA; (**B**) inhibition of invasion of PC3 cells by TUG1 siRNA; (**C**) knockdown of TUG1 reverses EMT in DU145 cells; (**D**) knockdown of TUG1 reverses EMT in PC3 cells. All tests were performed at least three times.

Next, Transwell assays showed the number of DU145 and PC3 cells in lower section were significantly reduced in the TUG1 knockdown groups compared with the control groups, which indicated that up-regulation of TUG1 promote cell invasion and metastasis (*P*<0.05; Figure 4A,B). Because EMT is vital for cell invasion, we next examined whether silencing TUG1 expression inhibited mesenchymal features. As expected, TUG1 knockdown decreased the expression of Vimentin and N-cadherin, and increased E-cadherin expression in DU145 and PC3 cells (*P*<0.05; Figure 4C,D). Therefore, the inhibition of TUG1 in PCa cells changed the cell morphology from a mesenchymal to a more epithelial phenotype.

### TUG1 inhibited miR-26a expression in PCa cells

**Figure 5:**
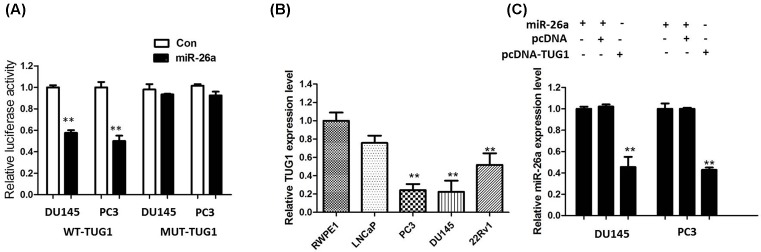
TUG1 inhibited miR-26a expression in PCa cells (**A**) The wild-type or mutant miR-26a-binding sites in TUG1 were inserted into pMIR-report luciferase vector. Luciferase activity was detected in PCa cells co-transfected with miR-26a or negative control (miR-control) and reporter plasmids containing WT-TUG1 (wild type) or MUT-TUG1 (mutant type). The normalized luciferase activity in the miR-control group was used as the relative luciferase activity. (**B**) Expression levels of miR-26a in different PCa cell lines were determined by qRT-PCR. (**C**) The co-transfection of miR-26A and TUG1 by pcDNA3.1. The expression of miR-26a was detected by qRT-PCR. All tests were performed at least three times. Data were expressed as mean ± S.D.; **P<0.01.

To investigate whether miR-26a was involved in the inhibitory effect of TUG1 on PCa cells, we applied the online software starBase v2.0. The prediction showed that TUG1 contains binding sequences complementary to miR-26a seed regions. For further confirmation, we used the luciferase assay to detect the association between TUG1 and miR-26a. The results showed that overexpression of miR-26a reduced the luciferase activity of the pMIR luciferase reporter containing wild-type TUG1 (WT-TUG1) but not mutant reporter (MUT-TUG1) vector (Figure 5A). Next, we measured the levels of miR-26a expression in various PCa cell lines. As shown in Figure 5B, the expression of miR-26a was obviously decreased in DU145 and PC3 cell lines, indicating the opposite result to TUG1 expression. In addition, we cloned the TUG1 into pcDNA3.1 vector and co-transfected into DU145 and PC3 cells with or without miR-26a overexpression. The results showed that overexpression of TUG1 inhibited the miR-26a expression (Figure 5C). All these data demonstrated that TUG1 associated with the miR-26a and may function as a ceRNA.

## Discussion

Some studies have suggested that increased lncRNAs levels are essential for tumorigenesis in a variety of biological cellular processes and multiple cancers. Numerous new lncRNAs, including HOTAIR, MALAT-1, and GAS5 [14–17], regulates numerous cellular processes important for tumorigenesis and progression of PCa. TUG1 was originally reported to be up-regulated in response to taurine treatment of developing mouse retinal cells. Aberrant expression of TUG1 in various tumors is related to increased cell proliferation and invasion and reduced apoptosis [18]. The clinical impact and molecular mechanisms of TUG1 in PCa patients remain uncertain. To shed additional light on this issue, we conducted an analysis investigating the clinical significance of TUG1 in human normal and cancerous tissues from prostate, and PCa cell lines, as well as the role of TUG1 in the regulation of tumor cell proliferation and invasion *in vitro*.

Consistent with previous reports, we confirmed that TUG1 was overexpressed in PCa tissues. Silencing of TUG1 was found to significantly impair PCa cell proliferation and invasiveness, suggesting that TUG1 is a critical oncogene. Our results demonstrated that inhibition of TUG1 could potentially have a suppressive effect on the migratory and invasive ability of PCa cells by regulating EMT.

To investigate the mechanism underlying the migration process, we measured the protein level of EMT markers following down-regulation of TUG1 expression. EMT is a process defined by cells losing their junctions, repressing E-cadherin expression, and exhibiting increased cell mobility. We further identified that the expression of EMT-related markers was significantly altered following TUG1 knockdown. Thus, our data suggest that TUG1-positive PCa cells possessed invasive properties by regulating EMT.

LncRNAs could act as miRNA sponges to regulate expression of various miRNAs and their target mRNAs [19,20]. Inspired by the ‘ceRNAs’ regulatory network, we hypothesized that TUG1 may also serve as a ceRNA. Then we searched for the interactions of TUG1 and potential miRNAs. Through bioinformatics analysis and luciferase assays, we discovered that miR-26a could form complementary base on the full-length TUG1 transcript and induce translational repression using a RLuc-TUG1 reporter gene. Our study showed the miR-26a expression reduced in PCa tissues, and qRT-PCR analysis showed that miR-26a expression was inversely correlated with TUG1 expression in PCa. Moreover, ectopic overexpression of TUG1 inhibited the miR-26a expression.

In conclusion, we found that the inhibition of TUG1 with specific siRNA results in potent antitumor activity in PCa *in vitro*, indicating that TUG1 has the potential to be an oncogene for PCa. Our data highlight the important role of TUG1 in the treatment of PCa.

## Abbreviations

lncRNA: long non-coding RNA
PCa: prostate cancer
qRT-PCR: quantitative real-time PCR
si-TUG1: siRNA against TUG1
TUG1: taurine-upregulated gene 1
